# Current Concepts in the Etiology and Pathogenesis of Pectus Excavatum in Humans—A Systematic Review

**DOI:** 10.3390/jcm11051241

**Published:** 2022-02-24

**Authors:** Vlad Laurentiu David

**Affiliations:** Department of Pediatric Surgery and Orthopedics, “Victor Babes” University of Medicine and Pharmacy, 2 Eftimie Murgu, 300041 Timisoara, Romania; david.vlad@umft.ro; Tel.: +40-757023237

**Keywords:** pectus excavatum, etiology, pathogenesis, chest wall malformations

## Abstract

Pectus excavatum (PE) is the most common deformity of the chest wall and is characterized by the posterior depression of the sternum and the lower costal cartilages. To date, the etiology of PE in humans remains enigmatic. Several etiologic hypotheses have been proposed over the past two centuries. However, most of them have been scientifically dismissed and now have only historic value. In this systematic review, we assess scientific publications of the past two centuries addressing the issue of the origin of PE in humans. We present and discuss the histologic, genetic, biomechanical, and experimental scientific achievements that contributed to the clarification of its etiology and pathogenesis. With no clear consensus over the exact mechanism, most recent studies agree that the primordial defect leading the deformation of the anterior chest wall in PE is related to the costal hyaline cartilage structure and function. Further studies on this subject must be carried out. Genetic studies seem to be the most promising way to understand the exact mechanism of PE’s origin and pathogenesis.

## 1. Introduction

Pectus excavatum (PE) is the most common deformity of the chest wall and is characterized by the posterior depression of the sternum and the lower costal cartilages [1]. By limiting the intrathoracic space and displacing some of the intrathoracic organs from their natural position, the deformity may have consequences over the normal function of the cardiac or respiratory system [2]. PE may also be the cause of significant psychological distress for these patients [1,2]. Surgery remains the main therapeutic treatment of PE with more than 50 different surgical techniques performed in the past century [3,4,5]. To date, the etiopathogenesis of PE is not fully understood. Several etiologic hypotheses have been proposed in the past two centuries. However, most of them were scientifically dismissed and now have only historic value. With no clear consensus over the exact mechanism, most of the contemporary studies indicate that the origin of the disease is to be found in an ultrastructural disorder of the costal cartilage [6]. The main question is how these cartilage disturbances lead to the inward bending of the anterior chest wall. Some authors advocate for the overgrowing of the costal cartilages, while others reject this hypothesis and are in favor of a low cartilage structural strength [1,4]. There are also etiologic hypotheses once considered obsolete and now being reconsidered, at least in part. We conducted a systematic search of the scientific literature of the past two centuries for publications addressing the issue of the etiopathogenesis of PE. In various online libraries and databases, we found approximately 100 publications addressing this matter. In this systematic review, we summarize and assess the main achievements to date in understanding the origin of PE in humans. We present and discuss this subject from histologic, genetic, anatomic, and biomechanical perspectives.

## 2. The Historic Perspective

Pectus-like deformities were identified in skeletons from 10th century graves excavated in Budapest [7]. Bauhinus made the first written description of the deformity in the 16th century [8]. Several case series and case reports were published during the 19th century [2]. Most of the early etiology hypotheses dating from this period were based on the direct observation of individual cases. Eggel (1870) presumed in his report that the reason for the deformity was a weakness and an abnormal flexibility of the sternum caused by nutritional disturbance or by developmental failure [9]. Langer and Zuckerkandel (1880) believed that the chest deformity was due to an intrauterine compression of the chin [10]. Flesch (1873) and then Hagmann (1888) were the first to believe that the overgrowth of the ribs might be responsible for the depression of the sternum [11,12].

At the beginning of the 20th century, the first surgical procedures were performed. Meyer in 1911 removed the second and third deformed costal cartilages on the right side and analyzed microscopically the removed cartilage [13]. He identified an unspecific degeneration but did not link the histological findings with the pathogenesis of PE [13]. There were several other etiologic hypotheses during the era, including the sternum remaining in an embryonic position, syphilis, sequels after mediastinitis, upper airway obstruction, trauma, and occupational habits, but none of them received full support from the scientific community [14,15]. Rickets was suggested as a possible cause by Kelly in 1929 [16]. However, later studies disproved this hypothesis, as PE patients did not reveal any clinical or paraclinical cardinal signs of rickets [17,18]. The first association between PE and collagen diseases was made by Curschmann, in 1936, when reporting a case of arachnodactyly, pectus excavatum, and ectopic lenses [19].

The prevailing etiologic hypothesis of the first half of the 20th century was that of the excessive diaphragmatic traction causing the posterior displacement of the sternum [20]. Lincoln Brown postulated this hypothesis and presented a surgical procedure to release the diaphragm from its sternal attachments [21,22]. He based his hypothesis on the observations of Bauhinus, who first suggested that depression of the sternum was caused by a shortened diaphragm [21]. Brown (1951) linked the excessive diaphragmatic pulling to a neuromuscular imbalance and muscle hyperactivity, a process similar to the pylorospasm in infancy [17]. This hypothesis was further developed by Brodkin (1951), who presumed that a thorax with a chondrosternal deformity is associated with an abnormally developed diaphragm, particularly the anterior portion that develops from the septum transversum [23,24]. The anterior portion is deficient and tendinous, and, in small children and infants with a more elastic, mobile anterior chest wall, the contraction of such an abnormal diaphragm will pull back the chest wall [23]. According to Jackson et al. [25], there is an imbalance between the traction of the pectoralis muscle and the diaphragm, with carinatum deformity occurring when there are spastic or overstimulated pectoral muscles, and the funnel chest where weak muscles permit the stronger diaphragm to “overpower” the pectoral groups and induce the excavatum deformity [25]. This concept of a diaphragmatic origin for chest wall deformities persisted for several decades and received support from many notable surgeons of the period, including Mark Ravitch and Charles Lester [26,27]. By the mid-1950s, many surgeons noted that there were some inconsistencies with the diaphragmatic hypothesis. In most cases, the point of maximal sternal depression is more cranial than the insertion of the diaphragm, and there is no visible diaphragmatic ligament [28,29]. Furthermore, the surgical detachment of the anterior diaphragmatic insertion, the Brown procedure, did not correct the pectus deformity [29,30,31]. Finally, Mullard (1967) pleaded strongly against the diaphragmatic hypothesis by arguing that the traction force of the diaphragm was too mild to depress a normal structured thoracic wall, that the deformity is dynamic and not congenital, and that, if there were a contracted, fixed diaphragm, the hearth could not be displaced [32]. He pleaded in favor of the defect of the costal cartilage as the primarily etiologic factor for PE, suggesting that there was a growing disturbance affecting the normal structure and function of the costosternal junction [32].

The methods of surgical correction of PE were influenced by the etiopathogenic concepts of their time. Meyer, then Sauerbruch, were the first to report successful surgical repair of PE [13,33]. Brown, a partisan of the excessive diaphragmatic traction hypothesis, performed surgical detachment of the diaphragm muscle from its sternal attachments [20]. However, his surgical procedure proved to be ineffective and was abandoned [29,30,31]. In 1949, Mark Ravitch presented his surgical technique of correction of PE. The procedure consisted in bilateral excision of the costal cartilages, sternal osteotomy, and relocation of the sternum in the correct position. The procedure was performed through an anterior thoracic midline incision [26]. Even though Ravitch agreed with diaphragmatic etiologic theory, his procedure gained wide support and was, for almost 50 years, the main surgical procedure for PE correction. Numerous improvements and variation of the technique were developed (Table 1). A revolution in the surgical treatment of PE happen in 1998, when Donald Nuss reported his technique of minimal invasive repair of PE (MIRPE). MIRPE was revolutionary for the time because it involved thoracoscopic approach, minimal incisions, and no cartilage resection or sternal osteotomy. Based on the costal cartilage overgrowth hypothesis, the principle of the technique is the forced correction of the chest wall deformity by means of a convex steel bar introduced beneath the sternum [34]. The functional and esthetic results of this procedure are excellent and MIRPE is, nowadays, the main surgical procedure for correction of PE worldwide [4]. A procedure mainly preferred by plastic surgeons is the silicon implant procedure [35]. The defect is filled with a custom-prepared silicone implant [35]. There are also some additional options for correction of PE that did not reached wide acceptation: sternal turnover, elastic correction, or implants of magnets (Table 1).

In the second half of the 20th century, the diaphragmatic hypothesis became obsolete, and there was a paradigm shift in the etiopathogenesis of PE. Reports associating PE with collagen diseases such as Marfan syndrome had been available since the turn of the century, and based on this, several authors stated that a growing disturbance in the costal cartilages was the most probable cause for PE [11,12,29,48]. The overgrowth of the costal cartilage became the favorite etiologic hypothesis [48]. According to this hypothesis, costal cartilages grow excessively compared with the other components of the thoracic cage, pushing the sternum backwards or, less frequently, forwards, producing pectus carinatum [48,49]. Even though there was no direct evidence, it seemed the most probable cause for PE based on several factors: the frequent association of PE with collagen diseases, the costal cartilages were deformed and look longer than normal, there was no abnormal diaphragm, and portions of the costal cartilages needed to be excised to correct the deformity [29,48,49]. This shift from the diaphragmatic hypothesis to the costal cartilage hypothesis marked the passage into the modern era. Most of the research is now focused on finding the cartilage disturbances that lead to the deformity of the chest wall.

## 3. The Histologic Perspective

The current majoritarian perspective over the ethology of PE identifies an abnormality in the structure, function, and/or physical features of the costal cartilage as the etiologic factor for the chest deformation. We were able to identify a limited number of studies that assessed the structure and function of the costal cartilage in patients with PE (Table 2).

The costal cartilage is a hyaline cartilage covered by a perichondrium. Its structure consists of a predominant intercellular matrix and chondrocytes [50]. The cells lie in groups of two or more and are contained in cartilage lacunae within the matrix. The intercellular matrix is made up of a fibrous component (collagen fibers, mainly type II) and a mixture of proteoglycans (mainly agrecan) [50]. The collagens give the tissue the ability to resist tension, while negatively charged proteoglycans attract cations (mainly Na^+^) and water, providing the resistance to compression [50]. There is a close relationship between the chondrocytes and the matrix. Chondrocytes regulate the synthesis and metabolism of the matrix, while the matrix composition influences the activity of the cells [50]. The costal cartilage links the ribs to the sternum, providing both stability and flexibility to the chest wall, and are responsible for the endochondral growth of the ribs [50].

Geisbe et al. (1967) were the first to publish, in the late 1960s, the results of a histologic analysis of the costal cartilages removed from patients with PE during corrective surgery [51]. Their findings were suggestive of premature aging, namely, the disturbance of costal cartilages [51]. However, they were not able to link the cartilage disturbances to the deformation of the chest wall. Moreover, there was the question of whether these cartilage disturbances were due to a primary, specific, metabolic defect in the cartilage cell or a result of incorrect loading of the tissue [51,52,53].

The cellular component of the costal cartilages is, in most of the recent studies, un-remarkable. Some of the early studies reported quantitative and qualitative disturbances of the chondrocytes: aging-like modifications, an increased number in a single chondron, or large acellular areas inside the cartilage [54,56,57,72]. However, most of these studies were conducted in the 1980s and early 1990s, lacking proper control groups and modern assessment methods. Recently, Kurkov et al. (2018) revealed that, even though the structure of costal cartilage is fundamentally identical in PE and controls, hypolacunar zones are more common, and hyperlacunar zones are less common in PE and PC than in control groups [70]. According to Tocchioni et al. (2013), the structural modification of the cartilage, including the chondrocytes, suggest that the costal cartilage undergoes a morphological stabilization after deformation rather than being a primarily cause for the deformity [69]. Indeed, the different distribution of the lacunae found by Kurkov et al. (2018) is more likely caused by the asymmetric loading of the deformed costal cartilages. Most of the recent reports proved that there are no significant numerical or structural differences in the chondrocytes between PE and controls [63,66,67,68]. This does not necessarily mean that the cellular content of the costal cartilages does not play a role in the pathogenesis of the chest wall deformities.

Almost all histologic studies to date revealed disturbances in the composition and structure of the extracellular matrix of costal cartilages in patients with PE. It seems like the structure of the collagen II fibers is perturbed, so it decreases its stability and structural strength [58]. Sokolov et al. (1989) identified an increased number of unstabilized N-terminal ends of collagen I fibers conducive to a decreased stability in the collagen network within the costal cartilages [58]. Moreover, the collagen II fibers are unequally distributed within the deep zones of the costal cartilage [65]. This fact might have major significance for the pathogeny of PE since the collagen fibers (collagen II in particular) are the components of the cartilage responsible for the strength, physical integrity, and stability of the cartilage structures [50]. Indeed, Feng et al. (2001) confirmed that costal cartilages removed from children with PE during surgical repair exhibit reduced physical strength when submitted to mechanical stress (tension, compression, and flexure) [65]. Kulik et al. (2013), citing Borisova et al. (1993), stated that there is a serious violation of the post-translational modification of the collagen network in PE [6,63,64]. This assumption is based on the fact that there is a decrease in the urinary excretion of some collagen degradation products (oxyproline-containing peptides and free oxyproline). According to this group, there is a qualitative, non-quantitative defect in the collagen content of the costal cartilages in PE [6].

There is a direct correlation between the biomechanical proprieties of the cartilage and the water and ionic content [50]. The negatively charged proteoglycans trapped in the extracellular matrix between the collagen fibers attract water and cations. This causes them to swell, increasing the tension within the collagen network until equilibrium is reached. This gives the cartilage stability and resistance to compression forces [73]. While some studies did not reveal significant modifications in the glycosaminoglycans and proteoglycans contained in the costal cartilage matrix in patients with PE [65,69], others [68] found decreases in the degree of sulfation in these glycosaminoglycans. They assumed that this might be the reason for the decrease in the absorption capacity of water and, hence, the deterioration of the mechanical characteristics of the costal cartilage [68]. A decreased volume of immobilized water and perturbation of the oligoelement content were noticed in samples of costal cartilages from PE patients [55,60]. The content of magnesium and calcium is increased, while zinc is reduced in PE cartilages compared to controls [55].

Summarizing this section, there is no doubt that the normal structure and function of costal cartilages is deeply disturbed in patients with PE. There is wide variability in findings depending on the cartilage component analyzed or the method used. It is also not clear which disturbances are primary and which are consecutive to the deformation of the chest wall. While some researchers believe that the cause of PE is based on a hyperplastic growth of the costal cartilage [67], others believe that the degradation of the biomechanical properties of costal cartilage is responsible for the deformation of the chest wall [65]. However, it is not mandatory that the two hypotheses are mutually exclusive; both abnormal growth and disturbed biomechanical proprieties might play a role in the deformation of costal cartilages in PE.

Unfortunately, there is a severe limitation for future histologic studies involving costal cartilages from patients with PE. Since minimal invasive repair of pectus excavatum (MIRPE) is now the prevalent surgical procedure used for the correction of PE, open correction of PE, which involves costal cartilage surgical excision, from which samples for histologic analysis are available, is now rare [4,26]. This means that large cohort histologic studies are no longer available.

## 4. The Genetic Perspective

Coulson was the first to suggest in 1820 the implication of genetic factors in the etiology of PE [74]. He reported a family of three brothers with PE. Sainsbury reported a family where PE occurred in lineal descent through four generations, also suggesting an autosomal dominant inheritance [75]. Several other reports of familial-type PE were published during the 19th and early 20th century [76,77,78,79]. However, the exact pathway of inheritance is yet to be discovered. With the purpose to identify the inheritance of this disease, Creswick et al. (2006) analyzed the pedigree of 34 families in which PE was present in two or more members [80]. His results were divergent and inconclusive, while autosomal dominant inheritance was suggested in 14 families, autosomal recessive inheritance was suggested in 4, and X-linked recessive inheritance was suggested in 6, and 10 families had more complex inheritance patterns [80]. Moreover, most of the cases of PE were sporadic, and family aggregation could be identified only in 45% of the cases [81]. Horth et al. (2012) assessed the pedigrees and clinical features of 116 individuals from 56 families [82]. They found strong evidence of autosomal recessive, genetic control for PE and that regions of Chromosomes 5, 15, and 17 are relevant for linkage mapping [82]. This is consistent with the histologic studies revealing alterations of the structure of the costal cartilage in patients with PE because the genes affecting cartilage (including fibrillin and agrecan) are also found on these chromosomes [82]. Other inheritance mechanisms, e.g., X-linked and autosomal dominant, could not be excluded but were limited by a small number of cases, and the autosomal recessive mechanism could be superposed in these cases [82].

A significant observation was made when PE was linked to known genetic diseases, mainly those that involved connective tissue disorders [83]. In Marfan syndrome, a primary hereditary connective tissue disorder, PE is considered a clinical manifestation of the inherited fibrillin-1 deficiency [84,85]. PE and PE-like deformities could also be associated with more than 27 genetic conditions, such as Ehler–Danlos syndrome, Noonan syndrome, Poland syndrome, and Holt–Oram syndrome [83,86,87,88,89]. The etiology of Poland syndrome is related to an abnormality in the development of one of the subclavian arteries during the embryogenesis leading to hypo-perfusion and ischemia on the affected side [90]. Unlike this genetic disease, most of the PE cases are non-syndromic, meaning that the exact pattern of inheritance is more likely to be complex and multifactorial [81,83]. The common characteristics of the majority of these genetic disorders is that they all involve, to certain degrees, a faulty synthesis or structure of collagen [91]. Historically, this fact was significant in deciphering the origin of PE in humans, because it indicates the costal cartilage as the starting point of the disease. However, in this context, identifying the faulty gene or genes involved in the origin of PE is difficult. Gurnett et al. (2009) identified a locus on Chromosome 18q12.1–q12.2 that is most likely responsible for the autosomal dominant inheritance in a family with an increased frequency of PE and adolescent idiopathic scoliosis [92]. However, the identified linkage region on Chromosome 18q contains more than 30 genes, and they were not able to isolate any causative mutation [92].

Recently, a mouse model of PE associated with adolescent idiopathic scoliosis (AIS) was obtained by genetically deleting G protein-coupled receptor 126 (Gpr126) in cartilage [93]. Gpr126 is a member of the adhesion G protein-coupled receptors family and is widely expressed on stromal cells [94]. Gpr126 was shown to bind collagen IV and laminin-211, promoting cyclic adenosine monophosphate, and has been identified in genomic regions associated with adult height, more specially trunk height [95,96]. This is interesting because the association of PE with scoliosis and a tall, thin stature (marfanoid type) is well documented in humans [1,4]. Moreover, in the animal model of PE and AIS developed by Karner et al. (2015), the deformation of both the vertebral column and the sternum occurred in mice during rapid growth prior to sexual maturity [93]. This is also consistent with the natural occurrence and progression of PE in humans, where the deformity of the chest wall progresses significantly during the preadolescence growth spurt [1,2,4]. The exact mechanism on which Gpr126 acts to induce the deformation of the chest wall is yet to be discovered. The authors proposed a link between Gpr126 and Gal3st4, where Gpr126 deficiency results in the upregulation of Gal3st4 gene [90]. GAL3ST4 encodes galactose-3-O-sulfotransferase 4, an enzyme responsible for catalyzing the C-3 sulfation of galactoses in O-linked glycoproteins [97]. Wu et al. [98] identified the mutation of the GAL3ST4 gene in a four-generation family with PE that showed dominant inheritance. Alteration in the cartilage proteoglycan sulfation pattern is a major factor for reduced cartilage strength and instability [99], and, coincidently or not, the sulfation pattern of the proteoglycans in the costal cartilages of the patients with PE was found to be faulty [68].

Tong et al. (2020) performed a whole-exome sequencing in a family with four generations of dominant inherited PE and identified a heterozygous stop-gain variant mutation in the tubulointerstitial nephritis antigen gene (TINAG) [100]. The mutation was identified only in PE patients and was not found in the healthy members of the family [100]. TINAG is a protein-coding gene coding a basement membrane glycoprotein initially identified as a target of antibodies in some forms of immunologically mediated tubulointerstitial nephritis [101]. Diseases associated with TINAG include membranous nephropathy and interstitial nephritis, and the gene was found to be associated with cell adhesion and formation of the extracellular matrix [101,102]. The mutation of TINAG was influential on normal cell development, in vitro cell proliferation being significantly inhibited compared to control cells [100]. However, a direct link between TINAG and PE deformation of the chest wall could not be demonstrated.

In summary, there is strong evidence that PE has a strong genetic component. The disease may be inherited in up to 45% of cases and is most likely subject to an autosomal recessive inheritance mechanism [82,83]. The PE may be syndromic, and more than 27 genetic conditions could be associated with PE-type deformities of the chest wall. However, most of the cases of PE are non-syndromic [83]. The most recent studies suggest that there is no single genic or chromosomal defect responsible for PE and that the disease etiology is most likely multifactorial. Abnormalities of Chromosomes 5, 15, 17, and 18 were related to PE [82,83]. The GAL3ST4 gene and the TINAG gene were found to be mutated in patients with PE, and the deletion of the Gpr126 gene leads to PE and AIS in mouse models [93]. Nevertheless, genetic aspects of PE are consistent with the histologic aspects, meaning that both indicate that the causes of the PE in humans is related to the normal structure and function of the costal cartilages.

## 5. The Anatomopathological and Biomechanical Perspectives

According to Schamberger, PE is present at birth in many cases [3,103]. However, deformity of the chest wall does not significantly progress during early childhood and causes little or no concern [1,3,4]. The age of presentation is more common after the age of 10, when the chest usually exhibits a relatively sudden depression [4]. In most cases, the deformity of the chest wall progresses quite rapidly during the pre-pubertal growth spurt [4]. Moreover, after the age of 16, when skeletal growth has nearly or completely stopped, the progression of the deformation of the chest wall stops as well [1,2,3,4,81,104]. On the other hand, most children with PE are tall for their age, thin, and often have a kyphotic posture, the so-called Marfanoid phenotype [104]. Notable for the etiology of the disease is the fact that several other syndromic or non-syndromic anomalies involving the hyaline cartilages are often found in patients with PE: scoliosis, congenital hip dysplasia and dislocation, congenital foot anomalies, Marfan syndrome, Ehlers–Danslos, Noonan, and neurofibromatosis [105]. This means that PE is more of a progressive deformity than a congenital one and that the sternal depression is related to the growth of the thoracic cage.

**Figure 1:**
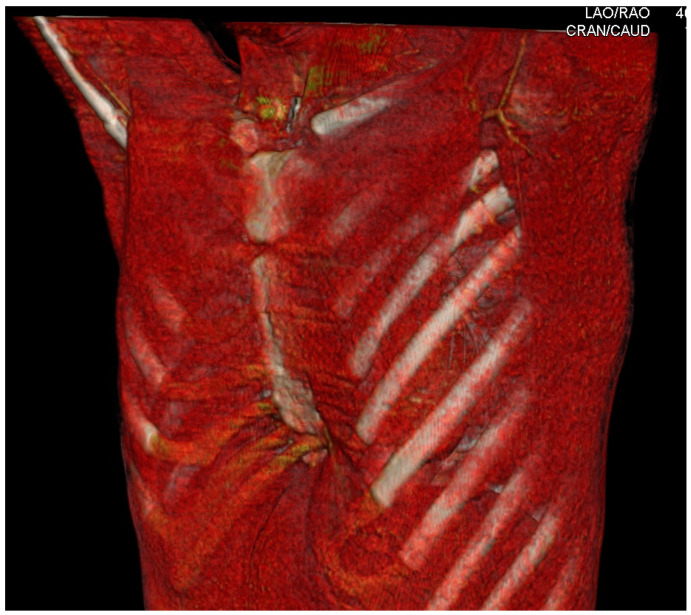
3D reconstruction of the CT images of the typical case of PE. Notice the posterior angulation of the lower part of the sternum and the adjacent costal cartilages. The deformation is symmetric.

The morphologic features of the chest wall deformity may be indicative of the origin of the disease. In typical cases of PE, the depression of the anterior chest wall most often includes the lower sternum and the adjacent costal cartilage [1]. The sternum is angulated posteriorly, just below the second costal cartilage insertion [106]. The lower costal cartilages, and sometimes the anterior part of the bony rib itself as well, follow the sternal bending and are also deformed, angulated towards the posterior (Figure 1) [106]. The defect may be narrow and focal, the so-called “cup-shaped” PE, or may be wide, the “saucer-shape” PE [1]. The severity of the depression is variable, from mild and barely visible to a severe and deep depression of the sternum, and it usually correlates with the age of the patients [1,3,4]. The chest wall deformity can be either symmetric or asymmetric. It is more often asymmetric, with the sternum rotated more frequently towards the right side [67].

Besides these morphologic features, several anatomic and physiologic factors have to be considered as well, in relation to the etiopathogenesis of PE: almost 75% of the longitudinal growth of a rib takes place at the sternal end, by endochondral bone formation from the costal cartilages [107]; the length of the rib arches and, among the adjacent costal cartilages, increases progressively from Ribs 1 to 7; there is an age-related growing pattern in the rib cage and there is a correlation with the number of cells at the costochondral junction in children [14]. These facts together lead to the conclusion that the perturbed growth of the lower costal cartilages is probably related to the depression of the sternum in PE patients [1,3,4]. This was the basis for the costal cartilage overgrowth etiology hypothesis [1]. However, the overgrowth hypothesis could not be clearly demonstrated. Besides the histologic and genetic studies, there are several imagistic studies assessing the morphology of the rib cage in relation to the origin of PE in humans. It seemed logical for several authors that, if the excessive growth of the costal cartilages is the cause of sternal depression, then the incriminated costal cartilages must be longer than normal [108,109,110,111,112,113,114]. However, the results of these studies are rather divergent. Nakaoka et al. (2009) found that the ribs and costal cartilages on the side with a more severe depression are shorter or equal than those on the opposite side in asymmetric PE [106]. When comparing the lengths of the costal cartilages on CT images of PE to normal, Nakaoka et al. (2010), David et al. (2016), and Karakilic et al. (2018) found that there are no differences [109,110,111]. Besides this, Karakilic et al. (2018) found no significant correlation between the length of the costal cartilages and the severity of the PE, evaluated by the Haller index [111]. Moreover, Eisinger et al. (2019) found shorter than normal costal cartilages in patients with PE [112]. On the opposite side, Park et al. (2015) and Kondo et al. (2020) found longer than normal costal cartilages in PE patients [113,114]. Without saying which author is right, the current available scientific knowledge on this matter indicates that the costal cartilage overgrowth hypothesis remains controversial and that there is no clear scientific information to support it.

Recent studies hypothesize that the inward deformation of the anterior thoracic in PE may happen due to the growth disturbance of the sternum [115,116,117]. Both Haje et al. (1999, 2021) and Lee at al. (2017), assessed the shape of the sternum in patients with chest wall deformities versus normal [115,116,117]. They observed that the sternum in patients with PE reveal abnormal shape, posterior curvature, torsion, and sometimes relatively sudden angulation [117]. Based on these findings, they concluded that the developmental disturbances of the sternum might play a role in the etiopathogenesis of chest wall deformities, including PE [115,116,117]. They and advocated for further histologic studies of the sternal growth plates [117].

## 6. Experimental Studies and Animal Models of Pectus Excavatum

We were able to identify only a few experimental studies on the etiopathogenesis of PE. Geisbe et al. (1967) conducted an experimental study in which the costal cartilages of young dogs were surgically weakened. Consequently, a funnel-like deformity was induced at the weakened segment of the anterior chest wall [51]. The suggested mechanism, backed up by Mullard (1967) as well, is that a structural insufficient anterior thoracic wall collapse is due to intra-thoracic aspirating forces [32,51]. Three decades later, Feng et al. (2001) confirmed that there is an alteration in the physical strength of the costal cartilages in patients with PE [65].

Recently, animal experimental models of PE were developed [118,119]. Wang et al. (2017) induced PE-like deformity in rabbits by removing a segment of the fifth, sixth, and seventh costal cartilages in rabbits. Subsequently, a gradual depression of the sternum in a PE-like deformity was revealed [118]. David et al. (2020) produced, using a similar mechanism, a rat animal model of PE [119]. Both of these animal models confirmed that, by weakening the costal cartilages, the anterior chest wall collapses in a PE-like deformity [118,119]. Moreover, a more recent study by David et al. (2022) investigated whether the overgrowth of costal cartilages induce the PE-like deformation in an animal model [120]. They induced the overgrowth of the last three costal cartilages by locally administering IGF1 in rats and observed that, even when the costal cartilages exhibit excessive growth, PE-like deformation of the chest wall did not occur [120]. This is a direct confirmation that the overgrowth etiologic hypothesis for PE is not to be fully trusted and that there must be a more complex explanation for the occurrence of PE in humans [120].

The study of Karner et al. (2015), who obtained an animal model of PE in mice, is worth mentioning again in this section [93]. In addition to the identification of Gpr126 involvement in the etiopathogenesis of PE, their animal model may open new doors into PE-related experimental research [93].

Even though there are only a few experimental studies, their results seem to point in the same direction. The structural strength of the costal cartilages, meaning the weakness of the costal cartilages, is directly related to the PE deformation of the chest wall [50,93,118,119]. The overgrowth of the costal cartilages alone cannot explain the deformation of the chest wall in PE patients [120].

## 7. Overview of the Current Concepts Regarding the Etiopathogenesis of Pectus Excavatum

In the past several decades, significant progress has been made in deciphering the circumstances in which PE deformity in the chest wall occurs in humans. Most specialists now agree on certain aspects, while other aspects remain controversial (Table 3).

Summarizing these concepts, it may be stated that PE is a disease that is most likely inherited and that the underlying defect leading to the depression of the sternum is present at birth. However, this defect manifests itself later in life, usually during the period of rapid skeletal growth. The primordial defect leading the deformation of the anterior chest wall is related to the costal hyaline cartilage structure and function. In regard to this, there are two main currents: the overgrowth of the costal cartilage hypothesis, and the hypothesis of the chest wall collapse due to the weak costal cartilage. Even though recent histologic, imagistic, and experimental studies point to this last-mentioned hypothesis, it has not been fully demonstrated. There is most likely an intricate mechanism by which a faulty growing pattern and altered physical features of the costal cartilages work in conjunction to induce PE deformation of the chest wall.

## Figures and Tables

**Table 1:** Main surgical procedures for the correction of Pectus Excavatum.

Author	Surgical Procedure	Etiological Hypothesis	Fate
Meyer 1911 [13]	First successful repair of PE Unilateral costal cartilage resection	Costochondral origin	Further developed into Ravitch procedure
Brown 1940 [22]	Section of the sternal diaphragmatic insertion	Diaphragmatic traction	Abandoned
Ravitch 1949 [26]	Open correction Bilateral costal cartilage resection Sternal osteotomy Multiple improvements	Substernal ligament	Main surgical technique in 20th century Still in use
	Rebheim: stabilization by metal blade [36]		
	Welsch: preserves the perichondrium [37]		
	Haller: tripod fixation [38]		
	Fonkalsrud: reattachment of the divided cartilages [39]		
	Willital: trans-sternal stabilization bar [40]		
Wada 1961 [41]	Sternal turnover Complete sternal detachment	Cartilage Overgrowth	Seldom, still in use
			High complication rate
Allen et al. 1979 [35]	Silicon implant The defect is filled with a silicon implant	-	Seldom, still in use
			Purely esthetic value
Nuss et al. 1998 [34]	Minimal invasive (MIRPE) Thoracoscopic approach No cartilage resection	Cartilage Overgrowth	Gold standard
Isakov et al. 1980 [42] Harrison et al. 2007 [43,44]	Minimover—magnetic implant Substernal placement of a magnet Second magnet on external corset Correction by magnetic attraction	Cartilage Overgrowth	Seldom used Limited experience Poor results [45]
Weber et al. 2006 [46,47]	Elastic correction Minimal cartilage resection Insertion of elastic thoracic bar	Cartilage Overgrowth	Limited experience

**Table 2:** Findings of the histologic assessments of costal cartilage samples from humans with PE (only direct reports of own experimental work were included).

	Method	Findings	Literature Report
1	LM	Regressive-cataplastic transformations	Geisbe et al. 1967 [51,52,53]
	BCH	Increased activity of catabolic enzymes of mucopolysaccharides	
2	LM	Asbestiform degeneration and transformation zones	Tischer et al. 1968 [54]
3	BCH	Low Zn levels	Rupprecht et al. 1987 [55]
		Increased Mg and Ca	
	EM	Presence of vascular channels	
		Areas of normal chondrocytes alternating with areas of degenerative chondrocytes with long-spacing collagen	
4	LM	Large acellular zones, map-like areas, unmasked chondrin fibers, and “marrow” cavities.	Kuritsyn et al. 1987 [56]
		Signs of early aging	
5	LM	Chondrocytes increases within the single chondrons	Rupprecht et al. 1989 [57]
6	BCH	Mutation in the N-terminal region of alpha 1 chain of collagen I	Sokolov et al. 1989 [58]
		Decreased stability of collagen II	
7	BCH	Deteriorations in the structure of collagen I, resulting in a lower stability of collagen II	Sokolov et al. 1990 [59]
		Similar findings in a patient with Ehlers-Danlos syndrome and PE	
8	BCH	Increased content of collagen	Tsvetkova et al. 1988 [60]
		Decreased content of immobilized water	
		Premature aging of the cartilages	
9	BCH	Synthesis of collagen was lower in skin fibroblasts of patients with PE	Tsvetkova et al. 1990 [61]
		PE is a connective tissue disease	
10	BCH	High levels of pyridinoline	Prozorovskaya et al. 1991 [62]
		Modified soluble/insoluble collagen ratio	
		Increased percentage of endogenous collagenolysis compared to controls	
11	CHG	Normal hydroxylysylpyridinoline and lysylpyridinoline crosslinks in PE patients (abnormal in Ehlers–Danlos and Marfan)	Borisova et al. 1993 [63,64]
12	LM	Unremarkable cellularity	Feng et al. 2001 [65]
		Unequal and patchy staining	
	BCH	Decreased biomechanical stability of the cartilage	
		Diminished strength at tension, compression, and flexure	
	EM	Collagen fibrils distributed unequally and arranged irregularly in deep zones of the cartilage	
13	LM	Disturbances in endochondral ossification and a growth of costal cartilage	Serafin et al. 2013 [66]
14	LM	Greater cellularity, more variable cellular distribution, larger vessel clusters, and more frequent myxoid matrix degeneration and focal necrosis	Fokin et al. 2009 [67]
15	LM	Unremarkable chondrocytes	David et al. 2011 [68]
		Unremarkable collagen fibers	
		PE cartilages have a lower content of strongly sulfated mucopolysaccharides, indicating an immature pattern	
16	LM	Great morphologic and morphometric variability of the cartilage	Tocchioni et al. 2013 [69]
	LM	PE cartilage are almost exclusively alcianophilic	
	ICH	No differences between control and PE	
	EM	No specific findings for PE	
17	LM	Decreased number of cartilage lacunae in PE	Kurkov et al. 2018 [70]
		Decreased number of cartilage channels	
18	LM	Amianthoid transformation present in both PE and normal cartilages	Kurkov et al. 2021 [71]
		Amianthoid fibers are thicker collagen II fibers	
	ICH	Type II collagen present, I and III absent, no differences between PE, PC, and normal	
	EM	Thicker matrix fibers in PE samples	
		Higher incidence of amianthoid transformation areas in PE and PC	

LM: Light microscopy; EM: Electron microscopy; BCH: Biochemistry; CHG: Chromatography; ICH: Immunohistochemistry.

**Table 3:** Overview of the current facts involved in the etiopathogenesis of PE.

Agreed Upon	The disease may be present at birth, but it has an uneven progression through life.
	Rapid progression of the deformity occurs during the prepubertal growth spurt, while the deformation becomes stable in adulthood, suggesting a direct linkage between skeletal growth and PE.
	Familial aggregation of PE.
	Most PE is non-syndromic.
	PE is frequently associated with several genetic syndromes that involve defects of the hyaline cartilage.
	The structure and the physical proprieties of the costal cartilages in patients with PE are severely disturbed.
	The diaphragm muscle, excessive traction, or fibrous bands are not involved in the etiopathogenesis of PE.
	There is no relation between the metabolism of vitamin D and PE, meaning that rickets are not responsible for PE deformation.
Controversial	The results of the histologic studies are disparate and sometimes in contradiction with each other, with respect to the different eras and different methods of investigation.
	Structural weakness in the costal cartilages causes the collapse of the anterior chest wall, meaning that such weakness is an etiologic factor of PE.
	Overgrowing lower costal cartilages grow excessively and push the sternum backwards, meaning that such overgrowth is the etiologic factor of PE.
	The mechanisms of inheritance include autosomal recessive (most likely), autosomal dominant, and X-linked.
	The genetic defect leading to the PE deformation is related to Chromosomal 5, 15, 17, or 18.
	The shape and length of the costal cartilages are different among PE versus normal subjects.

## Data Availability

Not applicable.

## References

[1] Kelly R.E. (2008). Pectus excavatum: Historical background, clinical picture, preoperative evaluation and criteria for operation. Semin. Pediatr. Surg..

[2] Brochhausen C., Turial S., Müller F.K.P., Schmitt V.H., Coerdt W., Wihlm J.M., Schier F., Kirkpatrick C.J. (2012). Pectus excavatum: History, hypotheses and treatment options. Interact. Cardiovasc. Thorac. Surg..

[3] Shamberger R.C., Welch K.J. (1988). Surgical repair of pectus excavatum. J. Pediatr. Surg..

[4] Nuss D., Obermeyer R.J., Kelly R.E. (2016). Pectus excavatum from a pediatric surgeon’s perspective. Ann. Cardiothorac. Surg..

[5] Nicodin A., Boi A.E.S., Popoiu M.C., Cozma G., Nicodin G., Badeti R., Trailescu M., Adam O., David V.L. (2010). Preliminary results after Nuss procedure. Chirurgia.

[6] Kulik I.O., Plyakin V.A., Sarukhanyan O.O., Ignat’Eva N.Y., Poludov S.A. (2013). Etiology and pathogenesis of pectus excavatum in children. Traumatol. Orthop. Russ..

[7] Toth G.A., Bud A.B.L. (2001). Funnel chest (pectus excavatum) in 10–16th century fossil material. J. Paleontol..

[8] Bauhinus J. (1609). Observatio. Ioannis Schenckii a Grafenberg.

[9] Eggel C. (1870). Eine seltene Mißbildung des Thorax. Virchows. Arch. Path Anat..

[10] Langer H., Zuckerkandel E. (1880). Untersuchungen über den mißbildeten Brustkorb des. Herrn. JW Wien. Med. Zeit.

[11] Flesch M. (1873). Ueber eine seltene Missbildung des Thorax. Virchow. Arch. Path Anat..

[12] Hagmann (1888). Selten vorkommende Abnormität des Brustkastens. J. B Kinderheilkd..

[13] Meyer L. (1911). Zur chirurgischen Behandlung der angeborenen Trichterbrust. Berl. Klin. Wschr..

[14] Muller C. (1906). Zur Entwicklung des Menschlichen Brustkorbs, Morphol Jahrb.

[15] Ochsner A., Debakey M.E. (1938). Chonechondrosternon: Report of a case and review of the Iiterature. J. Tboracic. Surg..

[16] Kelley S.W. (1910). Surgical Diseases of Children: A Modern Treatise on Pediatric Surgery. Ann. Surg..

[17] Brown A.L., Cook O. (1951). Funnel chest (pectus excavatum) in infancy and adult life. Calif. Med..

[18] Maneke M. (1959). Untersuchungen zur Pathogenese der Brustkorbverformungen1. DMW Dtsch. Med. Wochenschr..

[19] Curschmann H. (1936). Über erbliche Arachnodaktylie. Nervenarzt.

[20] Saxena A.K. (2017). History of Surgical Repairs of Chest Wall Deformities in Saxena AK editor. Chest Wall Deformities.

[21] Brown A.L. (1939). Pectus Excavatum. J. Thorac. Surg..

[22] Brown A. (1940). Lincoln: Pectus Excavatum (Funnel Chest). Anatomic Basis: Surgical Treatment of the Incipient Stage in In-fancy; and Correction of the Deformity in the Fully Developed Stage. J. Thorac. Surg..

[23] Brodkin H.A. (1951). Congenital chondrosternal depression (funnel chest) Its treatment by phrenosternolysis and chondroster-noplasty. Dis. Chest..

[24] Brodkin H.A. (1953). Congenital Anterior Chest Wall Deformities of Diaphragmatic Origin. Dis. Chest.

[25] Jackson J.L., George R.E., Hewlett T.H., Bowers W.F. (1959). Pectus excavatum: Surgical experiences in thirty-four cases. Am. J. Surg..

[26] Ravitch M.M. (1949). The Operative Treatment of Pectus Excavatum. Ann. Surg..

[27] Lester C.W. (1950). Funnel chest: Its cause, effects, and treatment. J. Pediatr..

[28] Koop C.E. (1956). The Management of Pectus Excavatum. Surg. Clin. N. Am..

[29] Sweet R.H. (1944). Pectus Excavatum: Report of Two Cases Successfully Operated Upon. Ann. Surg..

[30] Lindskog G.E., Felton W.L. (1955). Considerations in the Surgical Treatment of Pectus Excavatum. Ann. Surg..

[31] Mahoney E.B., Emerson G.L. (1953). Surgical treatment of the congenital funnel-chest deformity. AMA Arch. Surg..

[32] Mullard K. (1967). Observations on the aetiology of pectus excavatum and other chest deformities, and a method of recording them. Br. J. Surg..

[33] Sauerbruch F. (1920). Die Chirurgie Der Brustorgane.

[34] Nuss D., Kelly R.E., Croitoru D.P., Katz M.E. (1998). A 10-year review of a minimally invasive technique for the correction of pectus excavatum. J. Pediatr. Surg..

[35] Allen R.G., Douglas M. (1979). Cosmetic improvement of thoracic wall defects using a rapid setting silastic mold: A special technique. J. Pediatr. Surg..

[36] Rehbein F., Wernicke H.H. (1957). The operative treatment of the funnel chest. Arch. Dis. Child..

[37] Welch K.J. (1958). Satisfactory surgical correction of pectus excavatum deformity in childhood; a limited opportunity. J. Thorac. Surg..

[38] Haller J.A., Kramer S.S., Lietman S.A. (1987). Use of CT scans in selection of patients for pectus excavatum surgery: A preliminary report. J. Pediatr Surg..

[39] Fonkalsrud E.W. (2008). 912 Open Pectus Excavatum Repairs: Changing Trends, Lessons Learned: One Surgeon’s Experience. World J. Surg..

[40] Saxena A.K., Willital G.H. (2007). Valuable lessons from two decades of pectus repair with the Willital-Hegemann procedure. J. Thorac. Cardiovasc. Surg..

[41] Wada J. (1961). Surgical correction of the funnel chest “sternoturnover”. West. J. Surg. Obstet. Gynecol..

[42] Isakov J.F., Geraskin V.I., Rudakov S.S., Vasiljev G.S., Gerberg A.N., Burinov G.M., Muho S.B., Kondrashin N.I., Besjadovskaja G.L. (1980). A new method of surgical treatment of funnel chest with help of permanent magnets. Chir. Pediatr..

[43] Harrison M.R., Estefan-Ventura D., Fechter R., Moran A.M., Christensen D. (2007). Magnetic mini-mover procedure for pectus excavatum: I. Development, design, and simulations for feasibility and safety. J. Pediatr. Surg..

[44] Jamshidi R., Harrison M. (2007). Magnet-mediated thoracic remodeling: A new approach to the sunken chest. Expert Rev. Med. Devices.

[45] Graves C.E., Hirose S., Raff G.W., Iqbal C.W., Imamura-Ching J., Christensen D., Fechter R., Kwiat D., Harrison M.R. (2017). Magnetic Mini-Mover Procedure for pectus excavatum IV: FDA sponsored multicenter trial. J. Pediatr. Surg..

[46] Weber P.G., Hümmer H.P. (2006). Die “neue” Erlanger Trichterbrustkorrektur—Minimalisierung eines bewährten Verfahrens [The “new” Erlangen technique of funnel chest correction—minimalization of a well working procedure]. Zentralbl. Chir..

[47] Schulz-Drost S., Luber A.M., Simon K., Schulz-Drost M., Syed J., Carbon R.T., Besendörfer M. (2018). Elastic stable chest repair and its hybrid variants in 86 patients with pectus excavatum. J. Thorac. Dis..

[48] Giem R.N., Paulsen G.A., Dykes J. (1961). Pectus deformities. Calif. Med..

[49] Lester C.W. (1957). The etiology and pathogenesis of funnel chest, pigeon breast, and related deformities of the anterior chest wall. J. Thorac. Surg..

[50] Cormack D.H. (2001). Essential Histology.

[51] Geisbe H., Buddecke E., Flach A., Müller G., Stein U. (1967). Biochemical, morphological and physical as well as animal ex-perimental studies on the pathogenesis of funnel chest. Langenbecks Arch. Chir..

[52] Müller G., Flach A., Geisbe H. (1967). Morphological studies on resected rib cartilage in funnel chest. Frankf. Z. Fur Pathol..

[53] Geisbe H., Mildenberger H., Flach A., Fendel H. (1971). The aetiology and pathogenesis of funnel chest. Prog. Pediatr. Surg..

[54] Tischer W., Leutert G. (1968). Morphologische Veränderungen der Rippenknorpel bei Trichterbrust (Morphologic changes of rib cartilages in funnel chest). Beitr. Orthop. Traumatol..

[55] Rupprecht H., Hümmer H.P., Stöss H., Waldherr T. (1987). Pathogenesis of chest wall abnormalities—electron microscopy studies and trace element analysis of rib cartilage. Zeitschrift Kinderchirurgie.

[56] Kuritsyn V.M., Shabanov A.M., Shekhonin B.V., Rukosuev V.S., Rudakov S.S. (1987). Pathohistology of costal cartilage and immunomorphologic characteristics of collagen in funnel chest. Arkhiv. Patol..

[57] Rupprecht H., Freiberger N. (1989). Light microscopic studies of the cartilage in funnel chest. A new view of the pathogenesis. Z. Exp. Chir. Transpl. Kunstl. Organe.

[58] Sokolov B.P., Sher B.M., Kozlov E.A., Tsvetkova T.A., Rudakov S.S., Del’vig A.A., Kalinin V.N. (1989). Changes in the structure of type I collagen and cross-links between type I and type III collagen chains in a patient with funnel chest. Vopr. Med. Khim..

[59] Sokolov B.P., She R.B.M., Kozlov E.A., Tsvetkova T.A., Rudakov S.S., Del’vig A.A., Kalinin V.N. (1990). Structural charac-teristics of collagens from the skin and rib cartilage of patients with Ehlers-Danlos syndrome type II. Vopr. Med. Khim..

[60] Tsvetkova T.A., Kozlov E.A., Rudakov S.S., Del’vig A.A. (1988). Extractability of collagen from the rib cartilage and skin in funnel chest in children. Vopr. Med. Khim..

[61] Tsvetkova T.A., Gorokhova T.A., Del’vig A.A. (1990). The effect of ascorbic acid on the collagen synthesis in skin fibroblasts of children with funnel chest. Vopr. Med. Khim..

[62] Prozorovskaya N.N., Kozlov E.A., Voronov A.V., Verovskii V.A., Delvig A.A. (1991). Characterization of Costal Cartilage Collagen in Funnel Chest. Biomed. Sci..

[63] Borisova N.V., Pokrovskaya A.Y., Zakharova E.Y., Krasnopol’skaya K.D. (1994). Analysis of collagen hydroxypyridinium crosslinks in samples of tissues and urine of patients with inherited connective tissue disorders. Connect. Tissue Res..

[64] Borisova N.V., Pokrovskaya A.Y., Zakharova E.Y., Krasnopol’skaya K.D., Poliudov S.A. (1993). Analysis of hydroxypyri-dine cross-links of collagen from human rib cartilage. Biull. Eksp. Biol. Med..

[65] Feng J., Hu T., Liu W., Zhang S., Tang Y., Chen R., Jiang X., Wei F. (2001). The biomechanical, morphologic, and histochem-ical properties of the costal cartilages in children with pectus excavatum. J. Pediatr. Surg..

[66] Serafin J., Swiatkowski J., Majkusiak R., Nowakowski P. (2003). 40-year experience in surgical treatment of congenital chest deformationsethio–pathogenesis, operative techniques and clinical results. Acta Chir. Orthop. Traumatol. Cech..

[67] Fokin A.A., Steuerwald N.M., Ahrens W.A., Allen K.E. (2009). Anatomical, histologic, and genetic characteristics of congeni-tal chest wall deformities. Semin. Thorac. Cardiovasc. Surg..

[68] David V.L., Izvernariu D.A., Popoiu C.M., Puiu M., Boia E.S. (2011). Morphologic, morphometrical and histochemical pro-prieties of the costal cartilage in children with pectus excavatum. Rom. J. Morphol. Embryol..

[69] Tocchioni F., Ghionzoli M., Calosi L., Guasti D., Romagnoli P., Messineo A. (2013). Rib Cartilage Characterization in Patients Affected by Pectus Excavatum. Anat. Rec. Adv. Integr. Anat. Evol. Biol..

[70] Kurkov A.V., Paukov V., Fayzullin A.L., Shekhter A.B. (2018). Costal cartilage changes in children with pectus excavatum and pectus carinatum. Arkhiv. Patol..

[71] Kurkov A., Guller A., Fayzullin A., Fayzullina N., Plyakin V., Kotova S., Timashev P., Frolova A., Kurtak N., Pau-kov V. (2021). Amianthoid transformation of costal cartilage matrix in children with pectus excavatum and pec-tus carinatum. PLoS ONE.

[72] Bardakhch’ian E., Chepurnoĭ G., Shamik V. (2001). Ultrastructural changes of child rib cartilage in different deformations of chest cells. Arkhiv. Patologii.

[73] Hall A.C., Horwitz E.R., Wilkins R.J. (1996). The cellular physiology of articular cartilage. Exp. Physiol..

[74] Coulson W. (1820). Deformities of the chest. Lond. Med. Gaz..

[75] Sainsbury H. (1947). Congenital Funnel Chest. Lancet.

[76] Eggel W. (1870). Eine seltene Missbildung des Thorax. Virchow’s Archiv..

[77] Williams C.T. (1872). Congenital malformation of the thorax: Great depression of the sternum. Trans. Path Soc..

[78] Grunenthal A. (1888). Ueber Trichterbrust.

[79] Herbst E. (1887). Zur Kasuistik der Trichterbrust. Deutsch. Arch. Klin. Med..

[80] Creswick H.A., Stacey M.W., Kelly R.E., Gustin T., Nuss D., Harvey H., Goretsky M.J., Vasser E., Welch J.C., Mitchell K. (2006). Family study of the inheritance of pectus excavatum. J. Pediatr. Surg..

[81] Goretsky M.J., Kelly R.E., Croitoru D., Nuss D. (2004). Chest wall anomalies: Pectus excavatum and pectus carinatum. Adolesc. Med. Clin..

[82] Horth L., Stacey M.W., Proud V.K., Segna K., Rutherford C., Nuss D., Kelly R.E. (2012). Advancing our understanding of the inheritance and transmission of pectus excavatum. J. Pediatr. Genet..

[83] Kotzot D., Schwabegger A.H. (2009). Etiology of chest wall deformities—A genetic review for the treating physician. J. Pediatr. Surg..

[84] Dietz H., Adam M.P., Ardinger H.H., Pagon R.A. (1993–2022). Marfan Syndrome.

[85] Loeys B.L., Dietz H.C., Braverman A.C., Callewaert B.L., de Backer J., Devereux R.B., Hilhorst-Hofstee Y., Jondeau G., Faivre L., Milewicz D.M. (2010). The revised Ghent nosology for the Marfan syndrome. J. Med. Genet..

[86] Antia A.U. (1970). Familial skeletal cardiovascular syndrome (Holt-Oram) in a polygamous African family. Heart.

[87] Allanson J.E., Roberts A.E., Adam M.P., Ardinger H.H., Pagon R.A. (1993–2020). Noonan Syndrome.

[88] Miklovic T., Sieg V.C. (2021). Ehlers Danlos Syndrome.

[89] Romanini M.V., Calevo M.G., Puliti A., Vaccari C., Valle M., Senes F., Torre M. (2018). Poland syndrome: A proposed classification system and perspectives on diagnosis and treatment. Semin. Pediatr. Surg..

[90] Bavinck J.N., Weaver D.D. (1986). Subclavian artery supply disruption sequence: Hypothesis of a vascular etiology for Poland, Klippel-Feil, and Möbius anomalies. Am. J. Med. Genet..

[91] Armon K., Bale P. (2012). Identifying heritable connective tissue disorders in childhood. Practitioner.

[92] Gurnett C.A., Alaee F., Bowcock A., Kruse L., Lenke L.G., Bridwell K.H., Kuklo T., Luhmann S.J., Dobbs M.B. (2009). Genetic linkage localizes an adolescent idiopathic scoliosis and pectus excavatum gene to chromosome 18 q. Spine.

[93] Karner C.M., Long F., Solnica-Krezel L., Monk K.R., Gray R.S. (2015). Gpr126/Adgrg6 deletion in cartilage models idiopathic scoliosis and pectus excavatum in mice. Hum. Mol. Genet..

[94] Hamann J., Aust G., Araç D., Engel F., Formstone C., Fredriksson R., Hall R., Harty B.L., Kirchhoff C., Knapp B. (2015). International Union of Basic and Clinical Pharmacology. XCIV. Adhesion G Protein–Coupled Receptors. Pharmacol. Rev..

[95] Paavola K.J., Sidik H., Zuchero J.B., Eckart M., Talbot W.S. (2014). Type IV collagen is an activating ligand for the adhesion G protein–coupled receptor GPR126. Sci. Signal..

[96] Gudbjartsson D.F., Walters G.B., Thorleifsson G., Stefansson H., Halldorsson B.V., Zusmanovich P., Sulem P., Thorlacius S., Gylfason A., Steinberg S. (2008). Many sequence variants affecting diversity of adult human height. Nat. Genet..

[97] Seko A., Hara-Kuge S., Yamashita K. (2001). Molecular cloning and characterization of a novel human galactose 3-O-sulfotransferase that transfers sulfate to gal beta 1—>3galNAc residue in O-glycans. J. Biol. Chem..

[98] Wu S., Sun X., Zhu W., Huang Y., Mou L., Liu M., Li X., Li F., Li X., Zhang Y. (2012). Evidence for GAL3ST4 mutation as the potential cause of pectus excavatum. Cell Res..

[99] Heinegård D., Oldberg Å. (1989). Structure and biology of cartilage and bone matrix noncollagenous macromolecules. FASEB J..

[100] Tong X., Li G., Feng Y. (2020). TINAG mutation as a genetic cause of pectus excavatum. Med. Hypotheses.

[101] TINAG Tubulointerstitial Nephritis Antigen. https://www.ncbi.nlm.nih.gov/gene?Db=gene&Cmd=ShowDetailView&TermToSearch=27283.

[102] Jakowlev K., Livshits G., Kalichman L., Ben-Asher E., Malkin I., Lancet D., Kobyliansky E. (2007). Search for hand osteoar-thritis susceptibility locus on chromosome 6p12.3-p12.1. Hum. Biol..

[103] Shamberger R.C. (1996). Congenital chest wall deformities. Curr. Probl. Surg..

[104] Pilegaard H.K. (2016). Growth and pectus excavatum: Is there a relation?. Eur. J. Cardiothorac. Surg..

[105] Schamberger R.C., Welch K.J., Holder T.M. (1986). Chest Wall Deformities in Ashcraft KW.

[106] Schamberger R.C., Grosfeld J.L., O’Neil J.A., Fonkalsrud E.W., Corab A.G. (2006). Congenital Chest Wall Deformities.

[107] Peltomäki T., Häkkinen L. (1992). Growth of the ribs at the costochondral junction in the rat. J. Anat..

[108] Nakaoka T., Uemura S., Yano T., Nakagawa Y., Tanimoto T., Suehiro S. (2009). Does overgrowth of costal cartilage cause pectus excavatum? A study on the lengths of ribs and costal cartilages in asymmetric patients. J. Pediatr. Surg..

[109] Nakaoka T., Uemura S., Yoshida T., Tanimoto T., Miyake H. (2010). Overgrowth of costal cartilage is not the etiology of pec-tus excavatum. J. Pediatr. Surg..

[110] David V.L., Cerbu S., Haragus H., Popoiu M.C., Stanciulescu C.M., Cozma G., Burlac O., Boia E.S. (2016). Costal Cartilages Do Not Overgrow in Patients with Pectus Excavatum. Med. Princ. Pract..

[111] Karakılıç A., Karaçam V., Ersöz H., Ağababaoğlu I., Ulugün F.I., Şanlı A. (2018). Determination of severity of deformity with rib length to costal cartilage length ratio in thorax deformities. Turk. J. Thorac. Cardiovasc. Surg..

[112] Eisinger R.S., Harris T., Rajderkar D.A., Islam S. (2019). Against the Overgrowth Hypothesis: Shorter Costal Cartilage Lengths in Pectus Excavatum. J. Surg. Res..

[113] Park C.H., Kim T.H., Haam S., Lee S. (2015). Rib overgrowth may be a contributing factor for pectus excavatum: Evaluation of prepubertal patients younger than 10years old. J. Pediatr. Surg..

[114] Kondo S., Takagi D., Osaga S., Okuda K., Nakanishi R. (2020). The costochondral length in patients with pectus excavatum is longer than that of the normal thorax. Pediatr. Surg. Int..

[115] Haje S.A., Harcke H.T., Bowen J.R. (1999). Growth disturbance of the sternum and pectus deformities: Imaging studies and clinical correlation. Pediatr Radiol..

[116] Lee C., Zavala-Garcia A., Teekappanavar N., Lee C., Idowu O., Kim S. (2017). Measurement of sternal curvature angle on patients with pectus excavatum. Pediatr Surg Int..

[117] Haje D.P., Teixeira K.O., Silva M., Volpon J.B., Mendlovitz P.S., Dolabela P. (2021). Analysis of sternal curvature patterns in patients with pectus and control. Acta Ortop Bras..

[118] Wang R., Yang W., Cai L., Yang J., Xiang X., Zhang C., Jiang Q., Yang Y., Chen Z. (2017). Establishment of a rabbit model of pectus excavatum. Int. J. Clin. Exp. Med..

[119] David V.-L., Ciornei B., Horhat F.-G., Amaricai E., Horhat I.-D., Hoinoiu T., Boia E.-S. (2020). Rat Animal Model of Pectus Excavatum. Life.

[120] David V.L., Stanciulescu M.C., Horhat F.G., Sharma A., Kundnani N.R., Ciornei B., Stroescu R.F., Popoiu M.C., Boia E. (2022). S: Costal cartilage overgrowth does not induce pectus-like deformation in the chest wall of a rat model. Exp. Ther. Med..

